# Infectious Diseases in People Who Use Drugs Introduction

**DOI:** 10.1093/ofid/ofaf557

**Published:** 2025-10-07

**Authors:** Laura Marks

**Affiliations:** Division of Infectious Diseases, Department of Medicine, Washington University School of Medicine, St. Louis, Missouri, USA

**Keywords:** People Who Use Drugs, Sexually Transmitted Infections, Hepatitis C Virus, Endocarditis, Xylazine

## INFECTIOUS DISEASES IN PEOPLE WHO USE DRUGS SPECIAL COLLECTION

Over the past 2 decades, there has been an unprecedented increase in infectious diseases among individuals with substance use disorders (SUDs). These include bloodborne viruses, such as HIV [1] and viral hepatitis [2], along with life-threatening invasive bacterial and fungal infections [3] and outbreaks of sexually transmitted infections (STIs) [4]. The syndemic framework [5 ] has emerged to characterize and address the interconnected and compounding nature of these overlapping epidemics, including acknowledgment of the impact of social determinants of health and structural factors [6]. In the absence of interventions informed by this framework, syndemics can exacerbate the reach, impact, and consequence of each component epidemic [7].

This *Open Forum Infectious Diseases* special collection highlights articles on the epidemiology of syndemic infections in people who use drugs (PWUD). It showcases the novel ways that researchers and health care teams (see names listed in parentheses throughout) are preventing, treating, and managing these infections—with an emphasis on integrated patient-centered services and multilevel interventions to address structural factors.

## HIV AND PREEXPOSURE PROPHYLAXIS FOR PWUD

The collection opens with a series of articles evaluating the epidemiology (Fujita) and connections among polysubstance use, social drivers of health, mental health symptoms, and treatment utilization in people with HIV (Avery). Project RETAIN (Metsch) investigated the impact of patient navigation and substance use treatment with motivational enhancement therapy and cognitive-behavioral therapy on viral suppression among people with HIV who use cocaine but found no differences in viral suppression by group. Despite the negative trial findings, the results suggest that novel approaches to integrated care models are needed to achieve viral suppression and more effectively address individual and environmental complexities present within this population.

Preventing new HIV infections among PWUD must be a priority to achieve global HIV targets of ending the HIV epidemic by 2030 [8, 9]. Oral preexposure prophylaxis (PrEP) [10] is effective and well tolerated [11]; however, many people who inject drugs (PWID) report multilevel barriers to adhering to daily oral PrEP (McMahan). Long-acting injectable (LAI) formulations have redefined the experience of PrEP, with bimonthly intramuscular injections and biannual subcutaneous injections approved in 2021 and 2025, respectively [12]. In this special collection, 2 groups assess interest and willingness to take LAI PrEP among PWID in San Francisco (McMahan) and Washington, DC (Li). Participants in the San Francisco study by McMahan et al who were currently participating in a daily oral PrEP adherence trial described barriers to daily oral PrEP that could be mitigated by LAI PrEP. The Washington, DC, study (Xi) identified that although PWID reported a high prevalence of behaviors placing them at risk for HIV acquisition, PrEP awareness and uptake were low. Both groups highlighted the pressing need to increase PrEP awareness and uptake in this population and identified distinct patterns in PrEP modality preferences that should be considered when expanding access to LAI PrEP among PWID.

## VIRAL HEPATITIS

Contributors also examine the epidemiology of blood-borne viruses among PWUD. Asan et al document the prevalence of HIV, hepatitis B virus, and hepatitis C virus (HCV) in patients receiving alcohol and SUD treatment in Turkey. In the United States, Elnaiem and colleagues explore HCV treatment access disparities within a well-resourced health care system, identifying lower treatment rates among Black individuals, those experiencing homelessness, and individuals with Medicaid or no insurance. While these inequities continue to perpetuate a disproportionate burden of disease in already marginalized populations, researchers elsewhere in the collection identify opportunities to implement low-threshold HCV treatment in nontraditional settings, ranging from inpatient hospitalizations (Denkins) to syringe service programs (Yoder) and mobile medical units (Ramers, Tarfa). A qualitative study (Finbråten) of providers’ perspectives of implementation of low-threshold HCV treatment services identified that uptake was often hindered by insufficient funding for essential outreach, unmet patient social needs, and complex insurance barriers, underscoring the need for ongoing investment into multidisciplinary integrated care models.

## SEXUALLY TRANSMITTED INFECTIONS

STIs are an often overlooked part of the syndemic of infectious diseases in PWUD, and the supplement addresses the ongoing increases in reported STIs among PWID, with original research by Nacht and colleagues on the prevalence of STIs and associated risk factors among PWID in the San Diego–Tijuana border region. Overall, the study was consistent with other estimates of STIs among PWID in the United States, reporting that 6% of participants tested positive for 1 or more bacterial STIs and calling for routine testing for STIs among PWID at least annually.

## INTEGRATED SCREENING PROGRAMS

Practical strategies to identify care gaps and implement the aforementioned infectious diseases screenings (HIV, viral hepatitis, STIs, and latent tuberculosis infection) are explored in additional research within the supplement (D’Ottavi, Ho, Dyer, Knodel, Tarfa). Ho and colleagues evaluate a natural language processing dashboard designed to identify veterans with evidence of injection drug use across 6 Veterans Health Administration facilities. The natural language processing dashboard efficiently identified PWID within the VHA, but despite high levels of engagement with mental health services, comprehensive infectious diseases screening was uncommon, with 74% lacking bacterial STI screening and only 1 individual in the study receiving HIV PrEP. Offering perhaps one explanation for these findings, Bailey et al explored internal medicine resident perceptions of the barriers and facilitators to offering these critical infectious diseases screening and prevention services to hospitalized PWUD. Many residents reported assessing risk of HIV and hepatitis C among PWID at a higher frequency than STI screening, and the majority never assessed eligibility for HIV PrEP, citing multilevel barriers to prescribing PrEP from the inpatient setting.

Researchers in the collection reported more success in integrating infectious diseases screenings and care into SUD spaces. Dyer et al describe a program to successfully leverage the inpatient setting of an SUD treatment program to screen and deliver care not only for HIV and viral hepatitis but also for STIs, latent tuberculosis infections, and immunization delivery. Mirroring these findings, Knodel et al describe a model for mitigating common barriers to care for PWUD by providing comprehensive infectious diseases services collocated within a trusted community-based harm reduction program. Pilot findings from the first legalized mobile retail pharmacy and clinic in the United States for infectious diseases treatment and prevention tailored to reach PWUD (Tarfa) similarly provided comprehensive screening, treatment, and opioid use disorder care to address health care gaps for underserved PWUD within the community. These studies reinforce the importance of bundled comprehensive screening that goes beyond HIV and viral hepatitis screenings to include STI screenings and PrEP, pairing testing with immediate access to treatment delivery and preventative services. However, continued expansion of the scope of health care access points for infectious diseases screenings and treatment will be needed to stem the tide of the syndemic.

## INVASIVE BACTERIAL AND FUNGAL INFECTIONS

A conspicuous epidemic within the syndemic, bacterial and fungal infections in PWID can range from local skin and soft tissue infections to life-threatening invasive infections such as infective endocarditis (IE). This section begins with a comprehensive review of the breadth and severity of invasive fungal infections among PWID (Gonzales), along with a detailed exploration of the drug-drug interactions that can occur between antifungals and illicit drugs. This is followed by a description of an emerging outbreak of shigellosis in people experiencing homelessness and opioid use disorder in Philadelphia (Stedman).

To understand the risk behaviors and prevalence of bacterial skin and soft tissue infections and abscesses, Olson et al characterized injection practices associated with elevated risks for abscesses among 2 cross-sectional samples of PWID in Colorado, while Fanucchi and colleagues described injection-related infections and self-treatment practices among PWID in rural Appalachia. Elsewhere in the collection, authors used population-level data to explore the epidemiology of serious injection-related bacterial infections among US veterans with evidence of substance use (Harvey) and bacterial and fungal infections in New South Wales, Australia (Masters), and examine 5-year outcomes for PWID with IE in Southern Finland (Halavaara). The positive impact of concurrent SUD care (Jayasinghe, Masters) is a common theme in the management of invasive bacterial infections in PWUD. Research teams in this special edition approached this from multiple angles, evaluating patient perspectives (Jayasinghe), the accessibility of antimicrobial therapy and integrated SUD treatment options (Worden), and the impact of multidisciplinary care and planning for patients with serious injection-related infections in the United States (Douglass) and Scotland (Chung).

In a thought-provoking article, Rogers and colleagues use a US nationwide claims database to analyze 90-day readmission rates and central venous catheter events during outpatient parenteral antibiotic therapy for individuals with or without SUD, and they found that SUD was not independently associated with increased 90-day readmission or central venous catheter event risk, although it was a significant factor for overdose. To explore surgical management strategies for PWID presenting with IE, Purcell and colleagues analyzed outcomes in the TriNetX database for individuals with tricuspid valve IE who underwent percutaneous mechanical aspiration as compared with those who underwent traditional tricuspid valve surgery. After propensity matching between groups, there were no significant differences in short-term outcomes of death, heart block, or need for pacemaker implantation.

## XYLAZINE-RELATED INFECTIOUS COMPLICATIONS

The special collection concludes with a focus on the emerging complications of xylazine use (Berg, Yang). Xylazine is a potent centrally acting α-2 adrenergic agonist that has increasingly infiltrated the US illicit opioid supply over the past 5 years, leading to an emerging epidemic of necrotic skin wounds. Berg et al explore the microbiology of these infections, identifying that the predominate causative organisms are methicillin-resistant *Staphylococcus aureus* and β-hemolytic streptococci, with a small number of wound cultures also identifying *Pseudomonas aeruginosa* in individuals with associated bone and joint infections. Building on this understanding of the clinical microbiology of xylazine-associated wounds, Yang et al share multidisciplinary guidance to care for persons with xylazine-associated wounds.

## CONCLUSIONS

Collectively, these studies describe the clinical and public health burden of infections among PWUD. They illustrate that integrating syndemic care for PWUD into the practice of infectious diseases requires a coordinated, multifaceted approach that bridges research, education, and clinical care. We hope that the articles in this supplement catalyze conversations within the infectious diseases community and provide actionable insights that can be incorporated into developing guidance on best practices to transform routine clinical care for PWUD. At *Open Forum Infectious Diseases*, we welcome manuscripts on an ongoing basis that explore infections in PWUD to highlight potential solutions to improve health care access and outcomes in this population.
